# Family Government

**Published:** 1870-04

**Authors:** 


### FAMILY GOVERNMENT.

BY UNCLE BOB.

I sometimes think it is a very unfortunate thing to be a child. For a few years children seem to be the shuttlecock for every one. They are the great problem of the household and one which vexes it more than any other.

Just think of it old maids and bachelors. What do you think of having four or five, or even more little faces to wash; eight or ten hands to be scoured clean; as many stockings to be put on and shoes to be tied. Then the heads to be combed, dresses and aprons to be hooked or buttoned. Think of the multitude of cautions, hints, commands, not to say anything of the whippings which are daily called for by the necessities of the several individuals.

Here is one who must be ruled with a studious care, lest his persevering, impetuous zeal should be turned to an obstinate independence. The one who defines the distinction between *perseverance* and *obstinacy* as being only a difference between a determined *will* and a determined *wont*, was a shrewd observer of human nature. Rule the persevering zeal of a child and check his obstinate spunk, if you would make a strong, enterprising man, when grown to the age at which he must strike for himself.

But here is another child who is timid and over-sensitive to reproof. This one must be drawn out into every self-effort. It needs a parents tenderest care and sagacity, to develope the disposition of such an one. Examples must be constantly used as illustrating what you would attempt to make the child. Remember that such a child is not in the least inventive and should not be urged to break out into new paths. A judicious developement of this character, is sure to produce the most loving and lovable of dispositions. If it happens that the child of whom I speak is a girl, I will promise that she will be sought after, by the best of men when she reaches the age of womanly attractions.

Perhaps the family may have the worse of all forms of youthful torments, the selfish and naturally disobedient boy. A greater care and nerve is required in his guidance than in any other case.

The whole nature of the youngster must be changed. But few points of character will be apparent upon which the parent can lay hold. Review him as you will, the predominant knob wherever you would take hold, is self. He evinces a determination to rule all with whom he comes into contact, and to accomplish personal advantage and pleasure. If you as a parent wish to acquire the right to command and direct him, you will need the greatest perseverance. The conflict will be one of nature's, alone. If you are inclined to allow nothing to trouble you beyond a limited amount you will fail. There will be contests of hours, between you upon most trival things. As such they are of no importance in themselves—yet an easy surrender on your part, will make the next attempt to enforce your wish against his selfish desire, a matter of still greater difficulty. Be sure you *mean well* toward him when you check his desire. Show no passion or irritation in a petulant reproof, and persevere in whatever course you pursue. If tears will soften you, I advise you to have deliberated earnestly the occurrence of just such a case and have formed your decision as to a method of government. Do not rely upon the tenderness of your nature and allow it to be appealed to at an inauspicious moment. Exhibit your strength of purpose and conquer *fairly*. I say fairly because I have often seen a conflict closed and neither side knew which was the vanquished. Some round-about way had been resorted to by which a scene was prevented and peace of a sort was restored. Nothing is more absurd than this. I may say over-severity is less likely to occur than the opposite. I hope no one will accuse me of a lack of sympathy and say I am wanting in kindness. The full riot of a self-willed boy needs more than sugar plums and promises of a future punishment.

The *good* parent will abound in sympathy and yet be as inflexible as iron. A surgeon who amputates a limb, may not show his sympathy by unnerving himself, even though he is suffering in his own mind as he sees his knife separating the condemned and hurtful flesh.

The right of the parent to rule his or her house cannot be resisted. Neither should tears or anger be exhibited in the infliction of punishment. Resolve what you will do to promote the good of the family and check or encourage each member of it. Enlist the older children in your wish to render every one happy and useful. Do not however, allow the elders to rule or command by reason of their superior force or strength. Require all disputes to be settled only by your own rules, which rules must often be inculcated. I would say that to render the oldest child an acknowledged influencer, of the others, you can, in the presence of the others, refer some matter of the moment's interest, to that one and ask his or her opinion, and so far as you see good judgment in the reply you should allow it to be approved by you. Thus you can give force to good opinions and criticise whatever you consider faulty. The reliance of the younger in the wisdom of the older is strengthened, and you can be relieved of many minor responsibilities of government.

